# Physical performance and muscle strength rather than muscle mass are predictor of all-cause mortality in hemodialysis patients

**DOI:** 10.3389/fpubh.2023.1087248

**Published:** 2023-02-28

**Authors:** Xiaoyu Chen, Peipei Han, Kun Zhang, Zhenwen Liang, Chen Yu, Ningtao Lu, Zhouyue Shen, Fengyan Chang, Xin Fang, Qi Guo

**Affiliations:** ^1^Department of Rehabilitation Medicine, Shanghai University of Medicine and Health Sciences Affiliated Zhoupu Hospital, Shanghai, China; ^2^Department of Nephrology, Tongji Hospital, School of Medicine, Tongji University, Shanghai, China; ^3^Department of Rehabilitation Medicine, School of Health, Fujian Medical University, Fuzhou, China

**Keywords:** hemodialysis, muscle mass, muscle strength, physical performance, all-cause mortality

## Abstract

**Objectives:**

Patients undergoing maintenance hemodialysis usually suffer a high burden of poor functional status. The aim of this study was to investigate the association between muscle mass, muscle strength as well as physical performance with all-cause mortality in hemodialysis patients.

**Methods:**

923 hemodialysis patients (565 men, mean aged 61.3 ± 12.7 years) were included from eight facilities in Tianjin and Shanghai of China from 2019 to 2021. Muscle mass was evaluated by skeletal muscle index (SMI) and muscle strength was assessed by handgrip strength. Different measures of physical performance were measured *via* gait speed, Timed Up and Go Test (TUGT) and short physical performance battery (SPPB). Cox proportional hazards regression models were used to determine the adjusted hazard ratios (HRs) of mortality with 95% confidence intervals (95% CIs) for baseline muscle mass, muscle strength and different measures of physical performance. Additionally, the area under the Receiver Operating Characteristic (ROC) curves were constructed to determine which index is a better predictor of mortality.

**Results:**

During a median follow-up of 14 (12–17 months), 79 (8.6%) patients died. Using the Cox regression analysis, we founded that muscle strength and physical performance rather than muscle mass were significantly negatively associated with mortality. The C-index for different measures of physical performance in predicting mortality were 0.709 for SPPB, 0.7 for TUGT and 0.678 for gait speed, respectively. The C-index for muscle strength was 0.635, and the ability of prediction was significantly lower than the physical performance.

**Conclusions:**

Physical performance seems to a better indicator of mortality than muscle mass and strength in hemodialysis patients. Simple measures of physical performance may be appropriately used as a screening tool targeting high-risk hemodialysis patients for the prevention of mortality.

## Introduction

The prevalence of patients with end-stage renal disease (ESRD) is increasing rapidly, which has been a major public health problem in most countries (1, 2). Despite considerable improvement in dialysis modalities and patient care, hemodialysis patients still have an exceedingly higher mortality rate compared to the general population (3). The potential contributors to the poor survival status might be older age, comorbidities, malnutrition, underdialysis and decreased physical function (4, 5). It is reported that poor functional status is strongly related to advanced risks of adverse events in hemodialysis patients (6–8). Therefore, there is growing interest in finding effective and practical tests that can be used as screening tools to identify early populations that may benefit from targeted interventions.

Sarcopenia is a clinical disorder defined as loss of skeletal muscle mass and low muscle strength and/or physical performance (9). It's worth noting that muscle strength is not entirely dependent on muscle mass, and two elements may disassociate. With increasing age, muscle strength decreases at a rate greater than the rate of loss of muscle mass, even when muscle mass is maintained or increased (10, 11). As a result, there is a great interest in correctly distinguishing between the loss of muscle mass with muscle strength. Indeed, this is particularly important because treatments to maintain or increase muscle mass or muscle strength are not necessarily the same (12). Skeletal muscle plays a key role in metabolic function, facilitating glucose uptake and storage, and is related to physical performance. Recent studies suggest that muscle strength and physical performance were associated with mortality in hemodialysis patients (13–15).

However, they did not comprehensively take into account the various domains of physical performance indicators but only focused on one or two domains of physical performance, such as gait speed. Thus, it remains unclear whether or to what extent physical performance indicators are more independently associated to mortality in hemodialysis patients. In addition, several studies showed that higher muscle mass was independently associated with reduced risks of all-cause mortality in hemodialysis patients (16, 17), but previous findings regarding the relationship between the muscle mass and mortality have been discrepant (8, 18). Thus, more evidence is required to explore the associations between muscle mass, muscle strength and physical performance with mortality.

The purpose of this study was to identify the relationships between muscle mass, muscle strength and physical performance with mortality in patients on hemodialysis. Additionally, we aimed to examine which measure(s) was/were the most prominent in this relationship and could, accordingly, be relevant to be used in the clinical screening of hemodialysis patients. A broad understanding and addressing this are important for health care providers and policy makers in response to huge health care challenges.

## Methods

### Participants and study design

**Figure 1:**
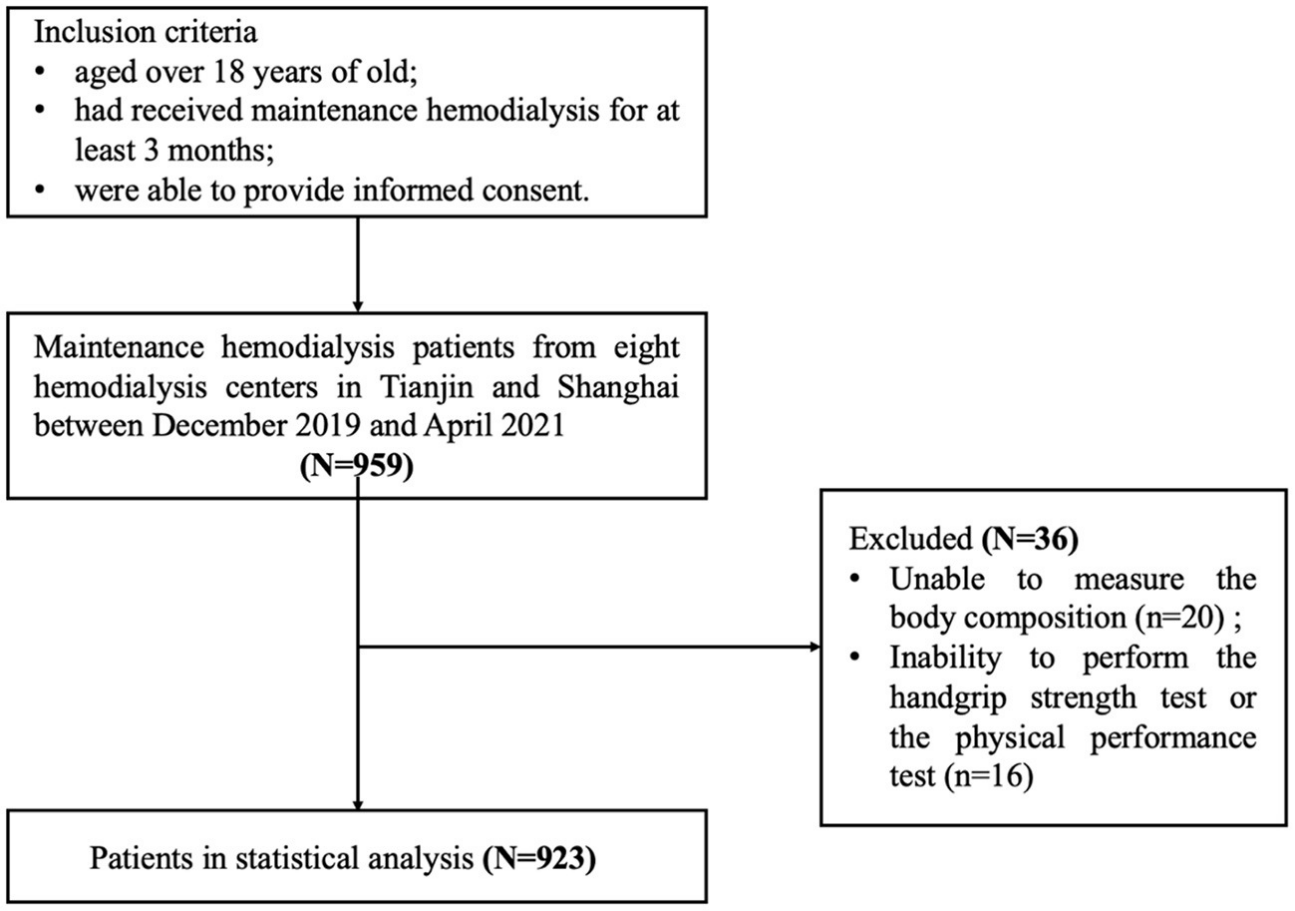
Flowchart of study participants.

This is a multicenter study including hemodialysis patients from eight hemodialysis centers in Tianjin and Shanghai between December 2019 and April 2021 at baseline. Patients were eligible to participate if they were over 18 years of age, had received maintenance hemodialysis for at least 3 months and were able to provide informed consent. Exclusion criteria was described as follows: (1) unable to measure the body composition; (2) inability to perform the handgrip strength test or the physical performance test; (3) patients with visual impairment or hearing impairment difficulties; (4) unable to communicate with researchers or provide informed consent. The final study sample comprised 923 subjects (Figure 1). All patients provided informed consent prior to enrollment in the study. This study was approved by the Ethics Committee of Shanghai University of Medicine and Health Sciences.

### Baseline variable

All patients were invited to a face-to-face interview to answer a standardized questionnaire. Socio-demographic characteristics (including age, gender, post-dialysis weight, dialysis vintage and education level), health behaviors (including smoking and drinking) and condition of chronic diseases were considered as covariates. The short form of the International Physical Activity Questionnaire (IPAQ) was used to assess the physical activity (19). Comorbidity was evaluated by the Charlson comorbidity index (20). All blood samples were drawn pre-dialysis. Details of measurement methods have been described in our recent study (21, 22).

### Assessment of muscle mass

Bioelectrical impedance analysis (BIA) (InBody S10; Biospace, Seoul, Korea) was used to measure muscle mass in the pre-dialysis period (23). Patients were placed in a supine position at least 10 min before assessment. Muscle mass was evaluated as the skeletal muscle index (SMI), calculated as the relative skeletal muscle mass index divided by the square of height (9).

### Assessment of muscle strength

A dynamometer was used to assess the muscle strength on the non-fistula prior to a dialysis session (GRIP-D; Takei Ltd, Niigata, Japan). Patients were asked to make maximum effort twice, and the result of the strongest handgrip strength was used in the analysis. For patients with indwelling dialysis catheters, we tested handgrip strength with the dominant hand.

### Evaluation of physical performance

Physical performance was measured *via* gait speed, Timed Up and Go Test (TUGT) and short physical performance battery (SPPB). We used the four-meter walking test to assess the gait speed. Patients were asked to stand and walk a distance of eight meters at a normal gait speed, and the time taken for the middle four meters was recorded. The patient was allowed to use a walking aid device (24). TUGT required a person to stand up from a chair, then walk 3 m, turn around, walk back and sit down again (25). The SPPB consists of three sequential tests that assess semi-tandem and tandem balance test, gait speed, and 5-sit-to-stand test. The total score ranges from 0 to 12 points, and 12 showing the best physical function performance to 0 showing inability to do the tests (26). All these physical performance tests were performed before a dialysis session.

### Statistical analyses

Baseline characteristics and clinical parameters are expressed as the means ± standard deviations (SDs) or as the numbers of patients and percentages. Analysis of independent *t*-test and chi-square test were used to compare variables between patients alive and deceased. Cox regression analyses are used to explore the association between muscle mass, muscle strength and physical performance with mortality, presented as hazard ratios with 95% confidence intervals. Kaplan-Meier curves were generated to assess the probabilities of the patient outcomes according to categorical of SPPB, and the Cox proportional hazards model was used for further multivariate adjustments with possible confounders including age, sex, BMI, IPAQ, Kt/v, albumin, hemoglobin, smoking, fall history, depression, malnutrition, and Charlson comorbidity index. The C -index was defined as the area under the ROC curves between individual measurement predictive probabilities for mortality. We further used C-index to identify the physical performance best correlated with all-cause mortality. Statistical analyses were performed using IBM SPSS Statistics v26.0 (SPSS Inc., Chicago, Illinois, United States). *P* < 0.05 was considered to indicate statistical significance.

## Results

### Baseline characteristics

The baseline characteristics of the study patients were presented in Table 1. Among the 923 patients (565 men), the mean age was 61.3 ±12.7 years. The median dialysis vintage was 45.12 months (range 22.19–92.80 months), and the mean BMI was 23.34 ± 3.82 kg/m^2^. Deceased patients were significantly older, and they tend to fall, depressed, malnourished and smoke (*P* < 0.05). Furthermore, BMI, IPAQ, handgrip strength, gait speed, TUGT, SPPB, hemoglobin levels, albumin levels and Kt/v were significantly lower in deceased patients than in patients alive (*P* < 0.05).

**Table 1:** Baseline characteristics of study participants according to the presence of mortality.

**Characteristics**	**Total**	**Alive**	**Deceased**	***P* value**
	**(*****n** =* **923)**	**(*****n** =* **844)**	**(*****n** =* **79)**	
Age (y)	61.3 ± 12.7	60.8 ± 12.7	67.0 ± 11.3	< 0.001
Male (%)	565 (61.2)	512 (60.7)	53 (67.1)	0.262
Dry weight (kg)	62.95 ± 12.88	63.18 ± 12.70	60.46 ± 14.48	0.072
BMI (kg/m^2^)	23.34 ± 3.82	23.44 ± 3.78	22.25 ± 4.10	0.008
Vintage (months)	45.12 (22.19, 92.80)	45.83 (23.13, 94.80)	39.2 (17.37, 70.13)	0.087
IPAQ (Met-min/wk)	1,386 (594, 3,066)	1,386 (693, 3,273)	660 (0, 1,533)	< 0.001
Education (%)				0.062
Less than high school	214 (23.2)	189 (22.4)	25 (31.6)	
High school or higher education	709 (76.8)	655 (77.6)	54 (68.4)	
Drinking (%)	11 (1.2)	11 (1.3)	0 (0.0)	0.421
Smoking (%)	200 (21.7)	180 (21.3)	20 (25.3)	< 0.001
SMI (kg/m^2^)	7.00 ± 1.21	7.02 ± 1.20	6.77 ± 1.35	0.077
Handgrip strength (kg)	24.81 ± 8.80	25.17 ± 8.74	20.93 ± 8.53	< 0.001
Gait speed (m/s)	0.97 ± 0.31	0.99 ± 0.29	0.79 ± 0.36	< 0.001
TUGT(s)	10.26 ± 7.90	9.76 ± 6.51	15.56 ± 15.77	< 0.001
SPPB	9.57 ± 2.96	9.80 ± 2.73	7.05 ± 4.04	< 0.001
Fall history (%)	329 (35.6)	288 (34.1)	41 (51.9)	0.002
Depression (%)	388 (42.0)	340 (40.3)	48 (60.8)	< 0.001
Malnutrition (%)	252 (27.3)	214 (25.4)	38 (48.1)	< 0.001
Number of medications (*n*)	4.49 ± 2.45	4.46 ± 2.44	4.72 ± 2.55	0.371
Charlson comorbidity index	3.87 ± 1.72	3.80 ± 1.71	4.58 ± 1.66	< 0.001
**Laboratory parameters**				
Hemoglobin (g/dL)	110.78 ± 16.29	111.25 ± 15.96	105.79 ± 18.92	0.004
Albumin (g/L)	39.41 ± 3.61	39.59 ± 3.44	37.47 ± 4.66	< 0.001
PTH (pg/dL)	356.86 ± 329.28	361.83 ± 330.38	300.76 ± 313.36	0.129
Calcium (mg/dL)	2.28 ± 0.26	2.27 ± 0.26	2.31 ± 0.31	0.270
Phosphorus (mg/dL)	1.93 ± 0.65	1.94 ± 0.64	1.88 ± 0.81	0.449
Kt/v	1.36 ± 0.33	1.37 ± 0.33	1.27 ± 0.29	0.013

BMI, body mass index; Kt/v, fractional clearance index for urea; SMI, skeletal muscle index; TUGT, timed up and go test; SPPB, Short physical performance battery; MIS, Mini Nutritional Assessment-Short Form; IPAQ, international physical activity questionnaire; Met-min/wk, metabolic equivalent task minutes per week; PTH, parathyroid hormone.

### Physical performance is associated with poor survival

**Figure 2:**
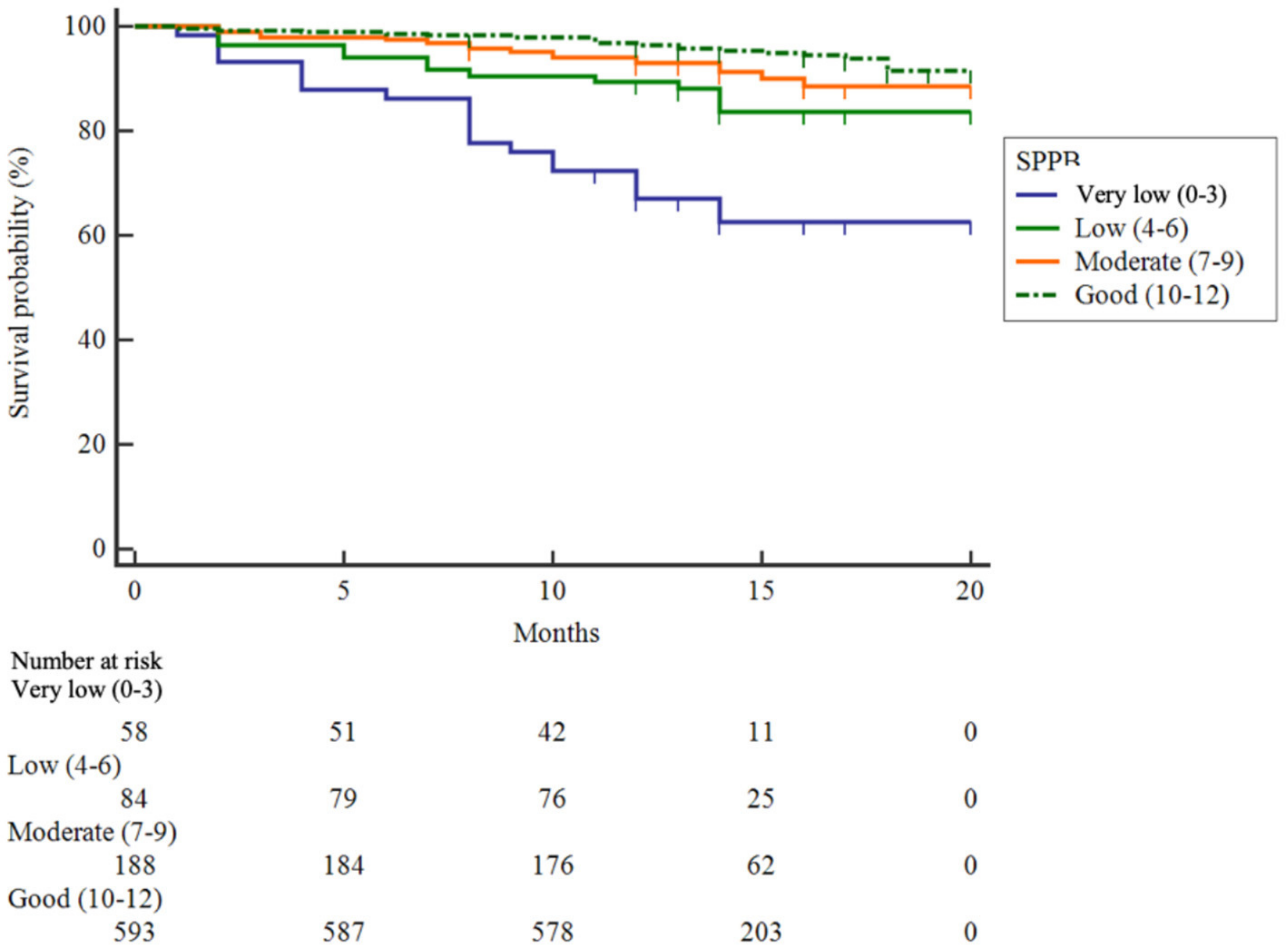
Kaplan Meier survival curves for all-cause mortality according to SPPB.

During a median follow-up of 14 months (10th percentile-90th percentile, 12–17 months), 79 patients (8.6%) died. The associations between muscle strength and different measurements of physical performance and mortality are presented in Table 2. After adjustments for potential confounders (age, sex, BMI, IPAQ, Kt/v, albumin, hemoglobin, smoking, fall history, depression, malnutrition, and Charlson comorbidity index), handgrip strength (HR = 0.96, 95% CI 0.92–0.99), gait speed (HR = 0.40, 0.18–0.92), TUGT (HR = 1.03, 1.01–1.04) were significantly associated with depressive symptoms. In the adjusted Cox regression model by SPPB categories, patients with very low (score 0–3) had significantly higher risks of death (HR = 3.98, 1.99–7.95). Survival curves of patients by SPPB categories are shown in Figure 2. Survival curves differed significantly at the log-rank test (*P* < 0.001).

**Table 2:** Cox regression model for the physical performance indexes as a predictor for the risk of mortality.

**Variables**	**Hazard ratio (95% CI)**			
	**Crude**	***P***	**Adjusted Model**	***P***
SMI	0.83 (0.69, 1.01)	0.058	0.86 (0.66, 1.14)	0.297
Handgrip strength	0.94 (0.92, 0.97)	< 0.001	0.96 (0.92, 0.99)	0.040
Gait speed	0.13 (0.07, 0.26)	< 0.001	0.40 (0.18, 0.92)	0.031
TUGT	1.04 (1.03, 1.05)	< 0.001	1.03 (1.01, 1.04)	0.001
**SPPB**				
Very low (0–3) (*n =* 58)	8.70 (4.93, 15.36)	< 0.001	3.98 (1.99, 7.95)	< 0.001
Low (4–6) (*n =* 84)	3.03 (1.55, 5.92)	0.001	1.61 (0.76, 3.41)	0.210
Moderate (7–9) (*n =* 188)	1.83 (1.01, 3.23)	0.046	1.32 (0.69, 2.51)	0.398
Good (10–12) (*n =* 593)	1		1	

Adjusted model: Adjusted for age, sex, BMI, IPAQ, smoking, falling history, depression, malnutrition, Charlson comorbidity index, hemoglobin, albumin and Kt/V. SMI, skeletal muscle index; TUGT, timed up and go test; SPPB, Short physical performance battery; BMI, body mass index; Kt/v, fractional clearance index for urea; IPAQ, international physical activity questionnaire.

### Predictive values of physical performance measurements for mortality

With regard to model discrimination, the C-index of the physical performance (C-index for TUGT: 0.678; SPPB: 0.709) were significantly higher than the muscle strength (C-index for handgrip strength: 0.635) (TUGT vs. handgrip strength: *P* = *0.046*; SPPB vs. handgrip strength: *P* = 0.020), and there was no significant between the handgrip strength and gait speed (C-index for handgrip strength: 0.635; gait speed: 0.678; *P* = 0.195, Table 3). However, there was no significant difference among the different measurements of physical performance in the values of C-index for mortality.

**Table 3:** Predictive values of muscle strength and physical performance measurements for mortality according to the C-index.

**Variables**	**C-index**	**SE**	***P***		
Handgrip strength	0.635 (0.603, 0.666)	0.0329	Ref		
Gait speed	0.678 (0.646, 0.708)	0.0332	0.195	Ref	
TUGT	0.700 (0.669, 0.729)	0.0314	0.046	0.293	Ref
SPPB	0.709 (0.679, 0.738)	0.0317	0.020	0.121	0.637

TUGT, timed up and go test; SPPB, Short physical performance battery.

## Discussion

This prospective cohort study demonstrated the influence of muscle strength and physical performance on the mortality risk in patients on maintenance hemodialysis, and supports the physical performance had superior prognostic discrimination for mortality, which deserved as an effective, costless and easily feasible screening strategy in this population. In contrast, no association was observed between muscle mass and mortality.

In line with our findings, recent studies suggest that muscle strength and physical performance were associated with mortality in hemodialysis patients (13, 14). These studies didn't comprehensively take into account the various domains of physical performance indicators but only focused on one or two domains of physical performance, such as gait speed. Our study also identified that very low physical performance in the SPPB, as well as gait speed and TUGT, were associated with lower survival and a higher risk of death. In addition, we observed that there was no association between muscle mass and mortality in our study, which was consistent with previous studies (8, 18). However, conflicting results have also been reported. Yajima et al. (16) and Fukasawa et al. (17) demonstrated that higher muscle mass was independently associated with reduced risks of all-cause mortality in hemodialysis patients. The main reasons for the differences are geographical differences and small population samples. In their study, the sample sizes were 162 and 81 respectively, which were significantly lower than the sample sizes of this study. Recent study has shown a strong association between sarcopenia and mortality in patients undergoing hemodialysis (27), so the association between muscle mass, one of the primary diagnostic factors for sarcopenia, and mortality needs to be further confirmed in the future.

Although the clinical relevance of muscle strength and physical performance as predictors of mortality in hemodialysis patients has been documented in previous studies, to date there have been no direct comparisons of the two tests. Interestingly, our results suggest that physical performance had superior prognostic discrimination for mortality than muscle strength. The possible reason may be that muscle function of lower extremities might be more important than that of the upper extremities regarding the patients' adverse outcomes. Previous research reported a discrepancy in upper and lower strength in a CKD cohort study (28), and other studies also revealed that muscle function in the lower extremities but not in the upper extremities was related to overall physical performance (29), suggesting the clinical importance of lower extremity performance. Furthermore, Johansen et al. found that physical performance such as gait speed declined frequently while handgrip strength didn't change over time among the hemodialysis patients (30). In that study, physical performance was the strongest predictor of mortality, which is similar to our results. Thus, we believe that monitor physical performance has the potential to be a valuable tool for continuous risk stratification of hemodialysis population.

Regarding the prognostic discrimination of different physical performance indicators for mortality, although SPPB showed the highest C index, it was not significantly different from gait speed and TUGT. The SPPB is an easy-to-apply instrument that includes balance, gait and lower strength, and has been used to evaluate the level of physical performance in different settings (31, 32). Our results show that patients with very low (score 0–3) by SPPB categories had significantly higher risks of death (HR = 3.98, 1.99–7.95). The cutoff points could help to identify hemodialysis with a higher risk of death at an early stage, given the easy applicability of the SPPB. Of note, SPPB includes three subtests, which would also take longer time to test than gait speed and TUGT. In the future, we need to consider whether isolated gait speed and TUGT would be useful to establish the predictive power for mortality, because there are advantages regarding time and costs to performing one test in an isolated manner compared to the entire SPPB.

Our study has several strengths. This is a multicenter study that comprehensively consider the association of muscle mass, muscle strength and various domains of physical performance indicators with mortality in hemodialysis population. In addition, most recognized confounders were taken into account into Cox regression models to analyze the independent association of physical performance and mortality in this study. Despite extensive efforts to curb study limitations, some limitations of this study should be considered. There is a concern about selection bias because patients who were incapable of performing the muscle strength and physical performance tests were excluded from our study. Second, our study was limited to Chinese patients on hemodialysis, thereby limiting the generalizability of our findings to the broader international hemodialysis population.

## Conclusion

Our study suggest that muscle strength and physical performance rather than muscle mass were significantly associated with all-cause mortality in hemodialysis patients. Furthermore, physical performance had superior prognostic discrimination for mortality than muscle strength, which is effective, costless and easily feasible screening strategy for better patient assessment and individualized care.

## Data availability statement

The datasets presented in this article are not readily available because our database is still expanding. All the multi-center hemodialysis centers have signed the data confidentiality agreement, so we are very sorry that we cannot upload it to the public database. But if you have any questions about the data, please write to us, and we will be happy to answer them for you. Requests to access the datasets should be directed to QG, guoqijp@gmail.com.

## Ethics statement

The studies involving human participants were reviewed and approved by the Ethics Committee of Shanghai University of Medicine and Health Sciences. Written informed consent to participate in this study was provided by the participants' legal guardian/next of kin. Written informed consent was obtained from the individual(s) and minor(s)' legal guardian/next of kin, for the publication of any potentially identifiable images or data included in this article.

## Author contributions

Study concept and design: XC, PH, and KZ. Acquisition, analysis, and interpretation of data: NL, ZS, FC, and XF. Drafting of the work: XC and PH. Critical revision of the manuscript: ZL, CY, and QG. All authors contributed to the article and approved the submitted version.
